# Identification and characterization of heteroclitic peptides in TCR-binding positions with improved HLA-binding efficacy

**DOI:** 10.1186/s12967-021-02757-x

**Published:** 2021-02-26

**Authors:** Beatrice Cavalluzzo, Concetta Ragone, Angela Mauriello, Annacarmen Petrizzo, Carmen Manolio, Andrea Caporale, Luigi Vitagliano, Menotti Ruvo, Luigi Buonaguro, Maria Tagliamonte

**Affiliations:** 1 Innovative Immunological Models Lab, Istituto Nazionale Tumori “Fond. G. Pascale”, Via Mariano Semmola, 1, 80131 Naples, Italy; 2 Institute of Biostructures and Bioimaging, CNR, Naples, Italy; 3 Present Address: Istituto Di Cristallografia-CNR, c/o area Science Park S.S. 14 Km 163.5 Basovizza, 34149 Trieste, Italy

## Abstract

The antigenicity as well as the immunogenicity of tumor associated antigens (TAAs) may need to be potentiated in order to break the immunological tolerance. To this aim, heteroclitic peptides were designed introducing specific substitutions in the residue at position 4 (p4) binding to TCR. The effect of such modifications also on the affinity to the major histocompatibility class I (MHC-I) molecule was assessed. The Trp2 antigen, specific for the mouse melanoma B16F10 cells, as well as the HPV-E7 antigen, specific for the TC1 tumor cell lines, were used as models. Affinity of such heteroclitic peptides to HLA was predicted by bioinformatics tools and the most promising ones were validated by structural conformational and HLA binding analyses. Overall, we demonstrated that TAAs modified at the TCR-binding p4 residue are predicted to have higher affinity to MHC-I molecules. Experimental evaluation confirms the stronger binding, suggesting that this strategy may be very effective for designing new vaccines with improved antigenic efficacy.

## Introduction

Tumor associated antigens (TAAs) are cellular self-antigens mostly overexpressed in cancer cells with low expression in normal cells. For this reason, they may be subject to both central and peripheral tolerance mechanisms leading to an inefficient immune response. Indeed, TAA-based cancer vaccines are well tolerated and have minimal side effects but have been proven to have limited efficacy in clinical trials [1]. Recognition of target non self-antigens by T cells is mediated by interaction between the T cell receptor (TCR) and the peptide-MHC-I complex (pMHC). In such interaction, the peptide is fastened to the MHC-I groove through the amino acid residues at the “anchor positions” while exposing the complementary “TCR-binding” residues to the TCR. Deciphering the different MHC-I binding motifs enabled the definition of the preferred peptide residues at the anchor positions for each HLA allele [2–5]. Heteroclitic peptides have been designed to enhance the stability of the pMHC-I complex as well as to increase the immunogenicity of the bound peptide [6–13].

An alternative approach for improving the immunogenicity of natural TAAs is to generate heteroclitic peptides mutated in the TCR-binding residues. The aim is to generate an epitope which is sufficiently different from the natural wild-type peptide presented by the cancer cells in order to break the immunological tolerance and induce a more potent CD8^+^ T cell response. However, such a difference should not be too extensive for the cellular immune response to retain the capability of cross-recognizing the natural structure and killing the presenting tumor cells [10, 11, 14–19]. Indeed, the low affinity between the TCR and the peptide-major histocompatibility complex (pMHC) allows the T-cell receptor to cross-react with multiple pMHCs [20–22].

In the present study, Trp2 and HPVE7 peptides, expressed by mouse melanoma B16F10 and TC1 tumor cell lines, respectively, were modified with different amino acid substitutions at the TCR-binding residue at position 4 (p4). Bioinformatics tools were used to predict and evaluate the impact of these amino acidic replacements on the antigen-MHC recognition. Moreover, experimental data demonstrated that even a mutation of a TCR-binding residue may increase the affinity for the MHC receptor. Based on these observations, we propose a rather simple strategy for developing vaccine antigens with improved antigenic efficacy.

## Materials and methods

### Prediction and design of heteroclitic peptides

Heteroclitic peptides for Trp2 and HPV E7 antigens were designed introducing each of the 20 genetically encoded amino acids at each position of the peptides. The predicted binding affinity of each heteroclitic peptide to the H2-Db was assessed by the NetMHCpan version 4.0 algorithm. Only mutations of the TCR-binding p4 residue (Tyr) predicted to have a higher affinity for the MHC compared to the wt peptide were selected for the experimental evaluation.

### Peptide synthesis

Individual peptides were synthesized at a purity > 95% determined by LC–MS analyses, as previously reported [23]. Lyophilized powder was dissolved in dimethyl sulfoxide (DMSO; Sigma-Aldrich), diluted in phosphate-buffered saline (1 × PBS; Gibco Life Technologies) and stored at − 80 °C until use.

### Peptide binding affinity assay

Peptide binding affinity to H2-Db molecule was performed for each selected peptide. Tap-deficient mouse derived H-2Db RMA-S cells were cultured for 24 h at 26 °C to accumulate empty MHC-I molecules on the cell surface [24]. Cells were washed in serum-free medium and incubated at 3 × 10^5^ cells/well with varying concentrations of peptides (1 μM, 5 μM, 10 μM) for 4 h at 37 °C with 5% CO_2_. Cells were harvested, washed with phosphate-buffered saline (1 × PBS; Gibco Life Technologies), stained with anti-H-2Db fluorescent monoclonal antibody (cat. 1157535; SONY) and analyzed by flow cytometry. All the experiments were performed in triplicate.

### Molecular docking of the E7 wt and Trp2 peptides and their heteroclitic variants with the H-2 class I histocompatibility antigen H-2Db

To perform molecular docking analysis of the complex between the major histocompatibility complex class I (MHC-I) H-2Db and the HPV-16 E7, Trp2 peptides and their heteroclitic variants, the structural data available for H-2Db was searched in the Protein Data Bank (PDB) (https://www.rcsb.org/). Although several structures of this murine MHC-I complexed with variety of peptides have been reported, no structural data is specifically available for the specific H-2Db/HPV-16-E7 as well as for H-2Db/Trp2 complexes. In this scenario, the PDB database was queried for complexes of H-2Db with peptides sharing some sequence similarity with HPV-16 E7 and Trp2 epitopes. A promising candidate was the PDB entry 1FG2 that corresponds to a complex of H-2D^b^ with the LCMV peptide with sequence KAVYNFATC [25]. The molecular modelling and docking analysis was performed by PyMOL and MolSoft molecular graphics systems.

## Results

### Prediction and design of heteroclitic peptides

**Fig. 1:**
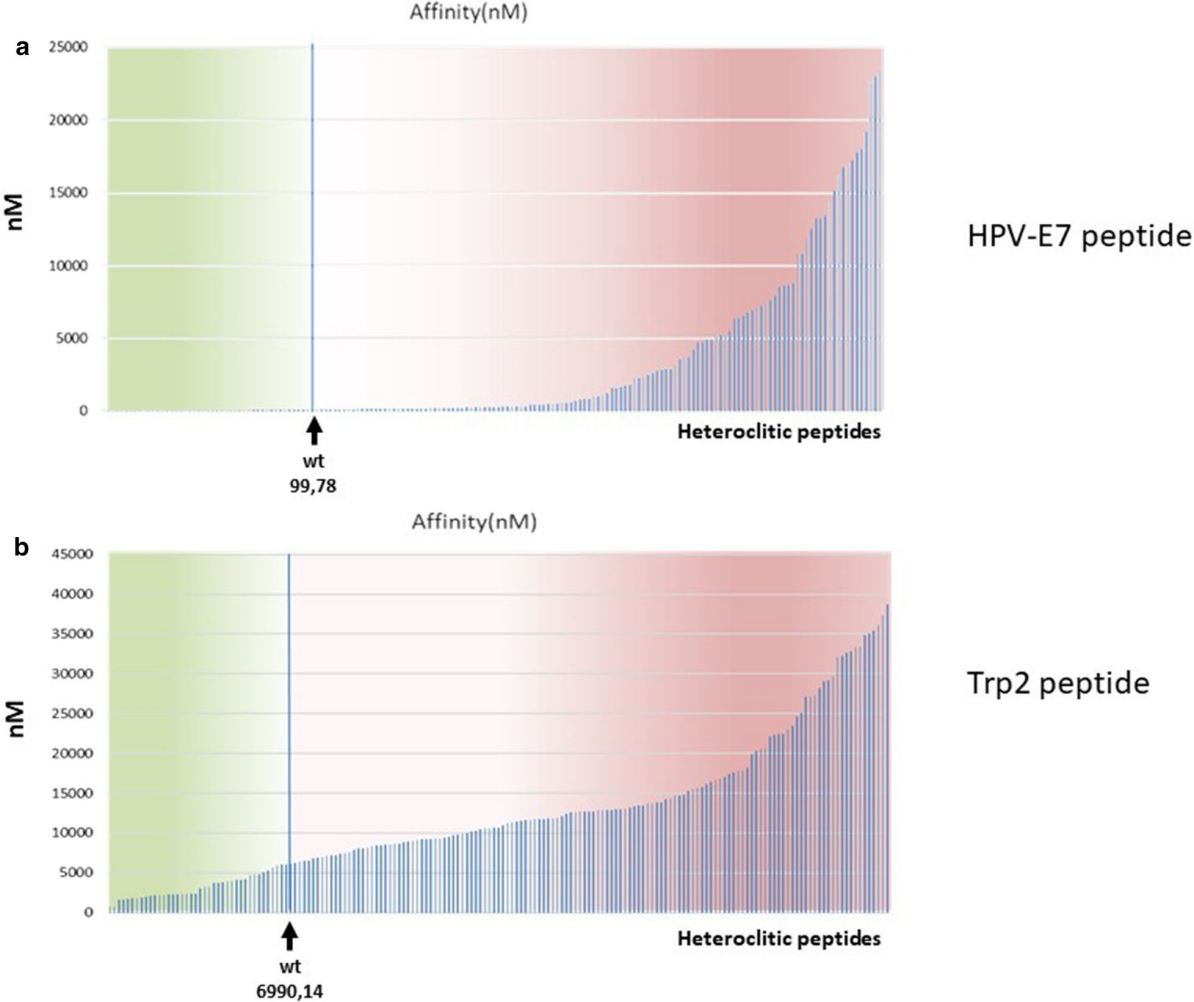
Prediction of binding affinity. **a**, **b** The predicted binding affinity to the H2-Db molecule of all possible heteroclitic HPV-E7 and Trp2 peptides is shown. The arrow indicates the affinity (nM) of the wt peptide. All the peptides with highest affinity (lower nM values) are on the left of the graph

An overall 171 (9 × 19) variants of the wild type sequence of Trp2 and HPV E7 epitopes were generated by placing in each position of the peptides all 20 amino acids leaving in the other positions the original residue. Prediction binding to the H-2Db major histocompatibility complex I (MHC-I) was obtained for all the heteroclitic epitopes with the algorithm NetMHCpan 4.0. Most of them were predicted to have a binding to H-2Db lower than the wt sequence, while a set of 47 (Trp2) and 45 (E7) heteroclitic epitopes showed a binding higher than the wt (Fig. 1a, b).

**Fig. 2:**
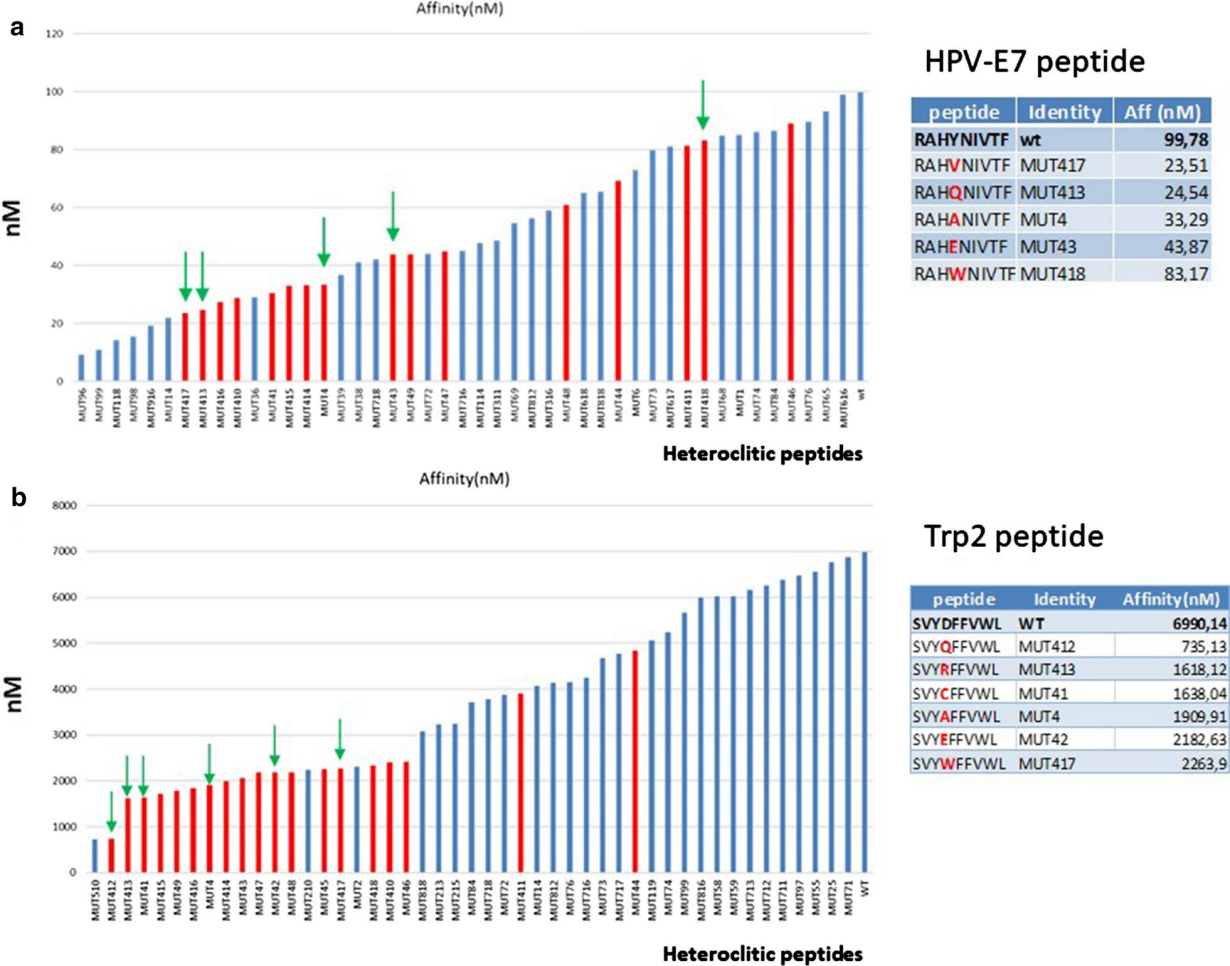
Peptide selection. **a**, **b** Heteroclitic peptides with affinity higher than the wt, with amino acid substitution in p4, are indicated for both HPV-E7 and Trp2 peptides. The ones selected for the present study are indicated by green arrow. The sequence and predicted binding affinity to H2-Db molecule of each peptide are shown

**Fig. 3:**
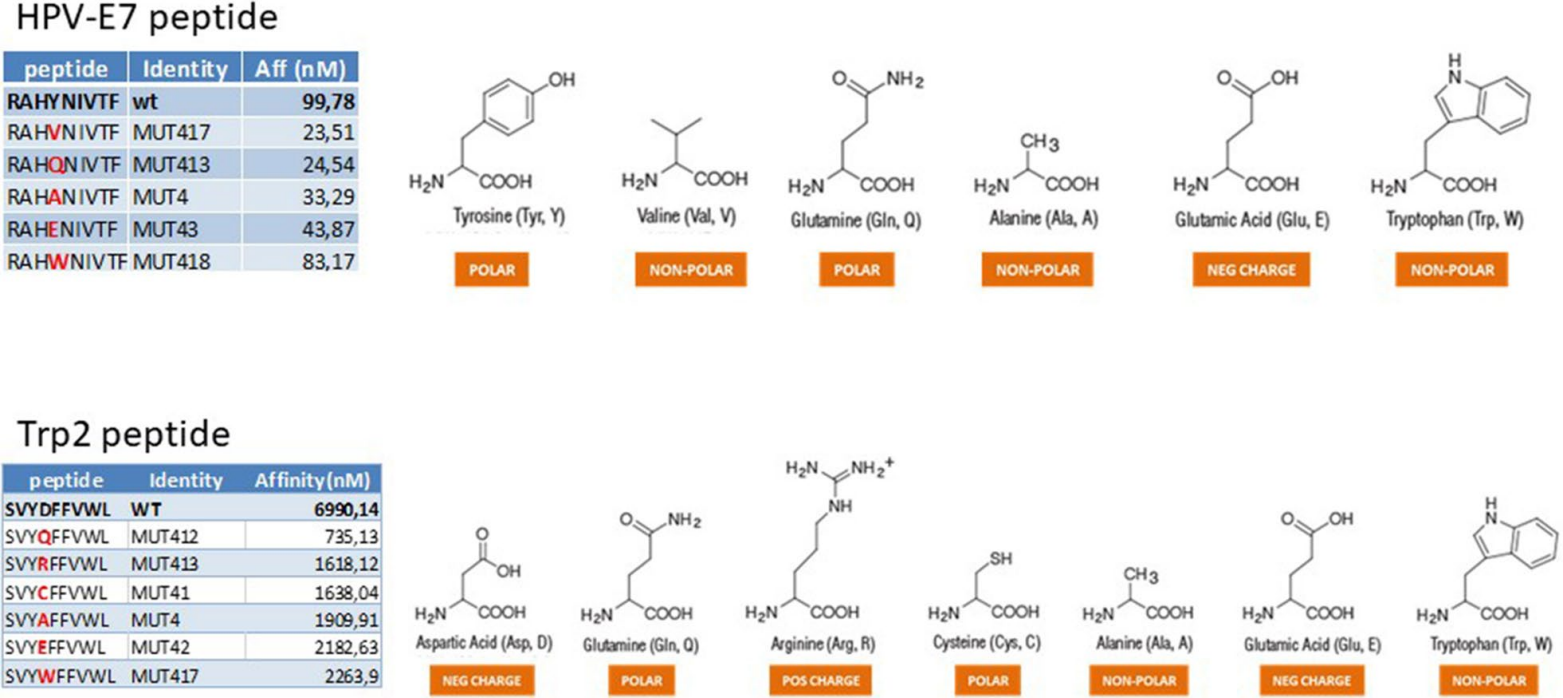
Structure of the amino acid residues at p4 in heteroclitic peptides. The structure of the substituting amino acid residues at p4 in the selected heteroclitic peptides is shown, together with the chemical properties of the side chains

Among the latter set of epitopes, those modified in the TCR-facing position 4 (p4) were selected for in vitro validation. Interestingly, the modification of the p4, which does not directly interact with the H-2Db molecule, had significant impact on the binding of the epitope. This was evident for both epitopes, but was more dramatic for the Trp2 which is a very poor binder in the wt form (6990 nM), and gained up to a 9.5-fold increase in the binding affinity to the H-2Db molecule (735 nM), although remaining a weak binder [17, 18] (Fig. 2a, b). In the HPV-E7 peptide, all the amino acid substitutions for the polar Tyrosine (Tyr, Y) were predicted to significantly improve the binding affinity to H2-Db molecule, regardless the chemical property of side chains. The best improvement was observed when the substitution involved either the non-polar Valine (Val, V) or the polar Glutamine (Gln, Q). The only exception was the non-polar Tryptophan (Trp, W) which did not induce a significant improvement in the binding affinity. Similar pattern was observed for the Trp2 peptide, and also in this case the best improvement was observed when the polar Glutamine (Gln, Q) substituted the negative charged Aspartic Acid (Asp, D) (Fig. 3).

### Structure modelling

**Fig. 4:**
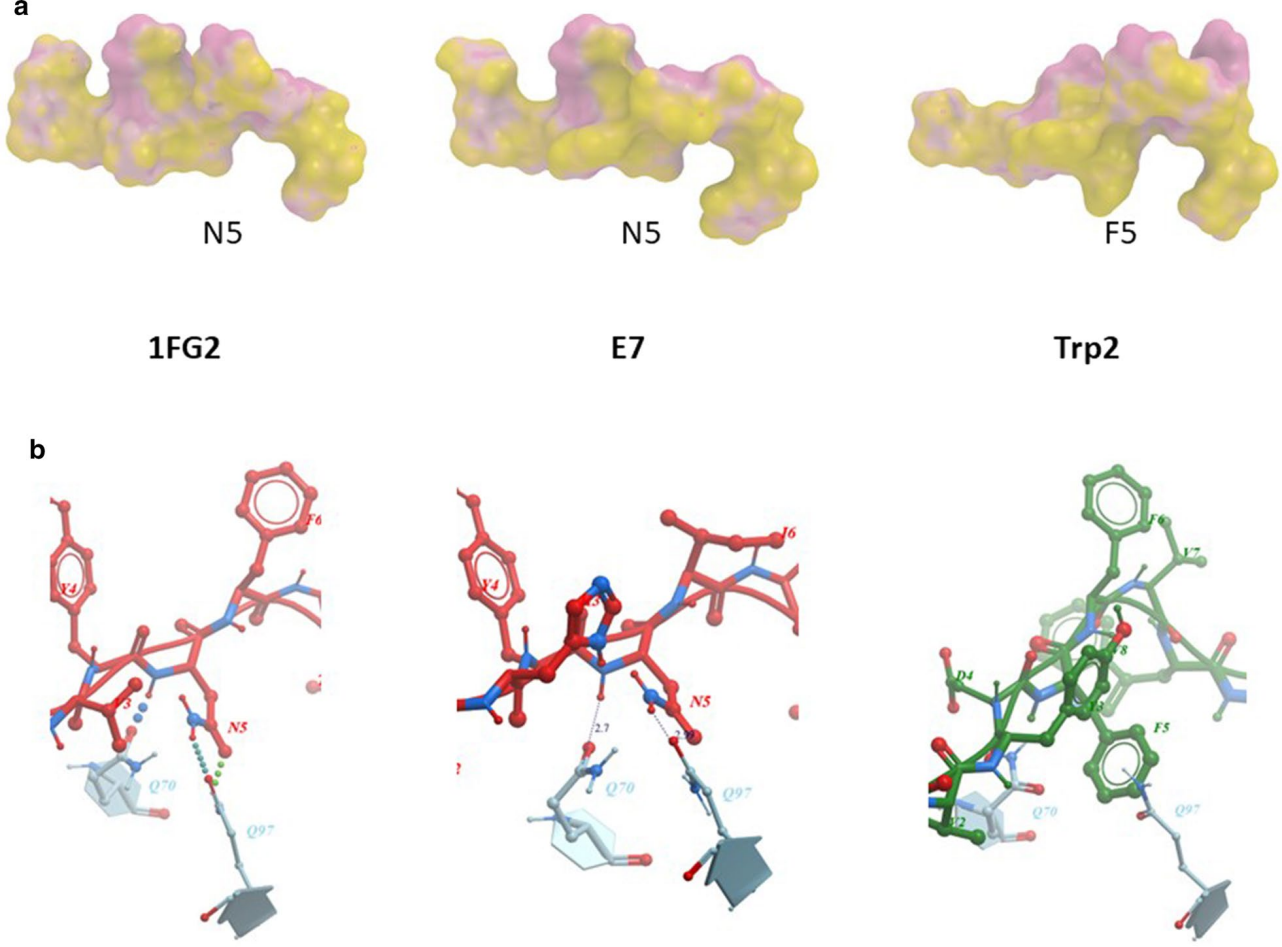
Predicted peptide conformation. **a**, **b** The conformation of the wt HPV-E7 and Trp2 peptides, bound to the H2-Db molecule, is shown compared to the LCMV peptide KAVYNFATC (1FG2, protein data bank). The amino acid at the anchor position 5 is highlighted for the three peptides. The presence of the non-polar Phenylalanine (Phe, F) in the Trp2 peptide results in a very poor binding to the H2-Db molecule, compared to the HPV-E7 and LCMV peptides

The impact of the amino acid substitutions on the structure of the heteroclitic peptides as well as the interaction with the H2-Db molecule, was assessed by structure modelling and molecular docking. We preliminarily modelled the complexes formed by the E7 and Trp2 peptides with H-2Db using the structure of these MHCI with the peptide KAVYNFATC (PBD entry 1F2) as template. Interestingly, the E7 epitope shares with the peptide of the complex an Asn residue in position 5 (N5) that represents a key anchoring point of the epitope on the H2-Db molecule. On the contrary, the weak binder Trp2 peptide has a totally different conformational structure as the side chain of the residue in position 5 (F5) could not be easily allocated in the H2-Db pocket (Fig. 4a). The latter observation is confirmed by the lack of hydrogen bond formation between the F5 of Trp2 and the Q70/Q97 residues of H2-Db which are those interacting with the N5 of 1FG2 and E7 peptides (Fig. 4b).

### Molecular docking

**Fig. 5:**
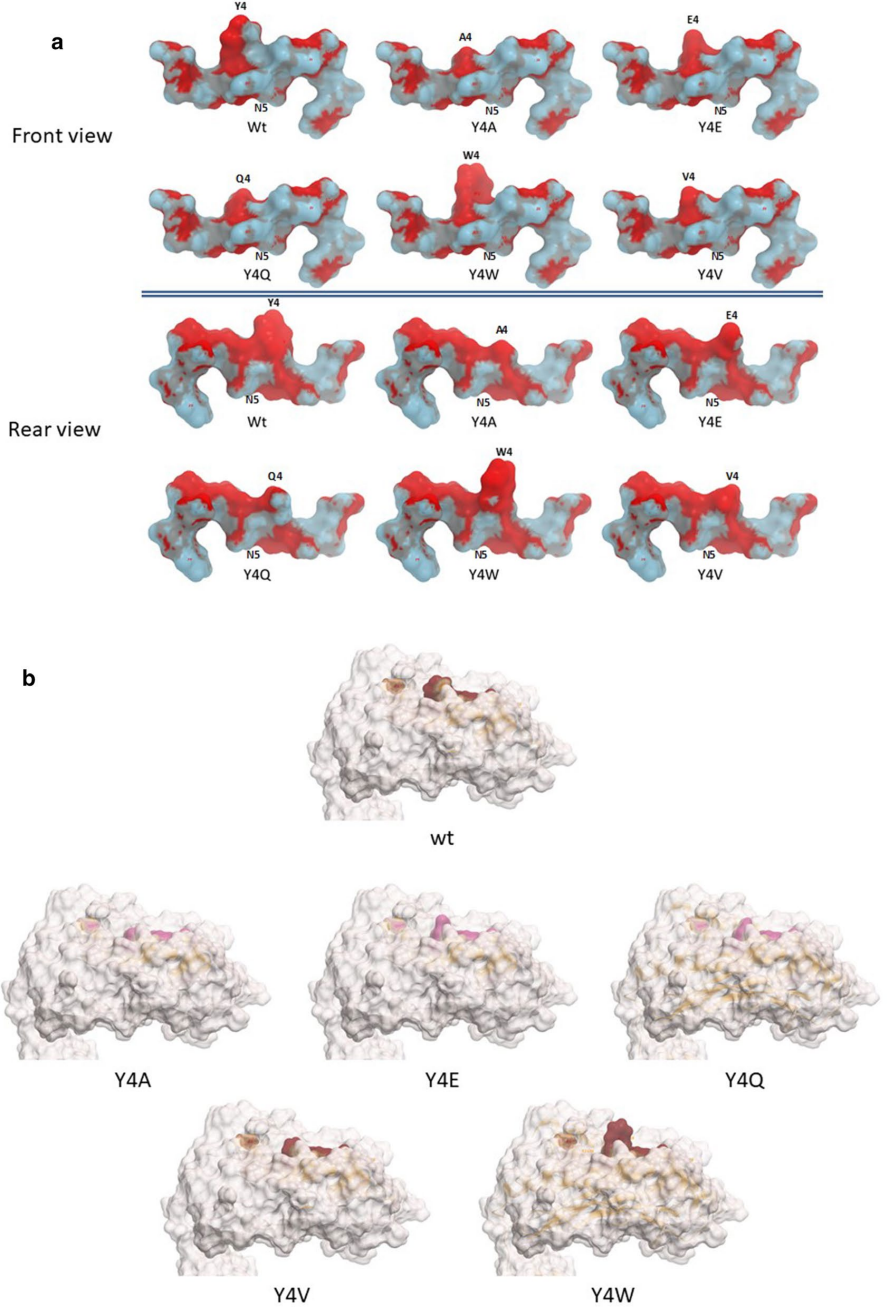
Predicted heteroclitic HPV-E7 peptides conformation. **a** The conformation of the heteroclitic HPV-E7 peptides, bound to the H2-Db molecule, is shown compared to the wt sequence. Contact pattern to the H2-Db are indicated in grey for both front and rear sides. **b** Prediction of the heteroclitic peptides’ structure presented to the CD8^+^ T cell for interaction with the TCR

In order to verify whether the substitutions of the residue in p4 do impact on the interactions with the H2-Db, molecular docking simulations were performed. The results show that none of the substitutions introduced in the p4 residue of the HPV-E7 peptide significantly changes the front as well as rear contact points of the E7 peptide with the H2-Db molecule. The only exception is the Y4Q substitution, which shows a loss of contact in the rear face at the p3/p4 tract (Fig. 5a). However, the amino acid pattern exposed to the TCR by each heteroclitic peptide, when docked into the H2-Db groove, shows significant differences compared to the wt. Indeed, only the Y4V substitution results in a conformation of the TCR binding residues very similar to the wt peptide (Fig. 5b).

**Fig. 6:**
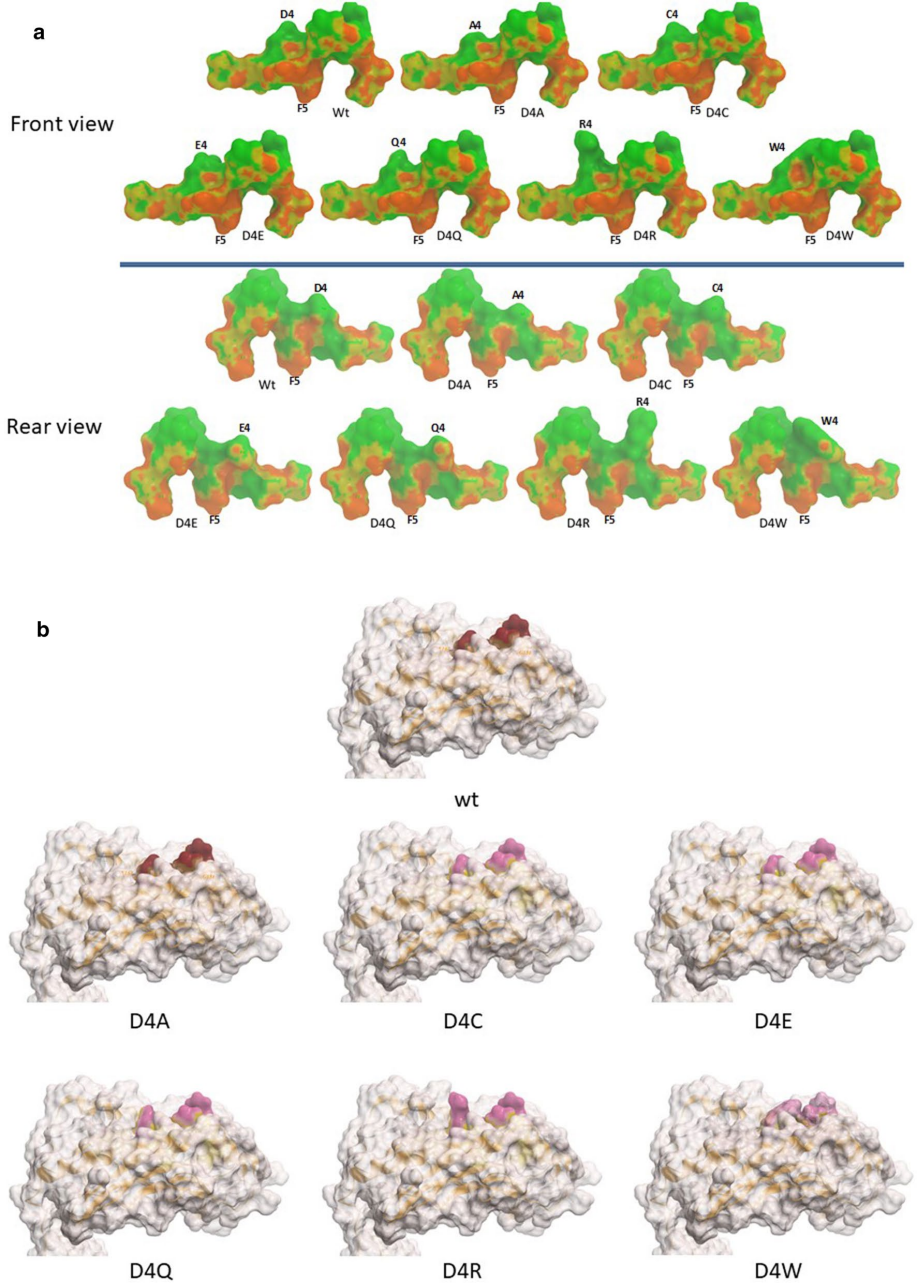
Predicted heteroclitic Trp2 peptide conformation. **a** The conformation of the heteroclitic Trp2 peptides, bound to the H2-Db molecule, is shown compared to the wt sequence. Contact pattern to the H2-Db are indicated in maroon for both front and rear sides. **b** Prediction of the heteroclitic peptides’ structure presented to the CD8^+^ T cell for interaction with the TCR

Likewise, all the substitutions introduced in the heteroclitic peptides of the Trp2 epitope show an identical pattern of contact points with the H2-Db of the wt peptide (Fig. 6a). Accordingly, the amino acid pattern exposed to the TCR by each heteroclitic peptide, when docked into the H2-Db groove, shows a substantial similarity compared to the wt. Indeed, only the D4R and the D4W substitutions result in a conformation of the TCR binding residues significantly different from the wt peptide (Fig. 6b).

### In vitro analysis of peptide binding affinity and stability to H-2 Db molecule

**Fig. 7:**
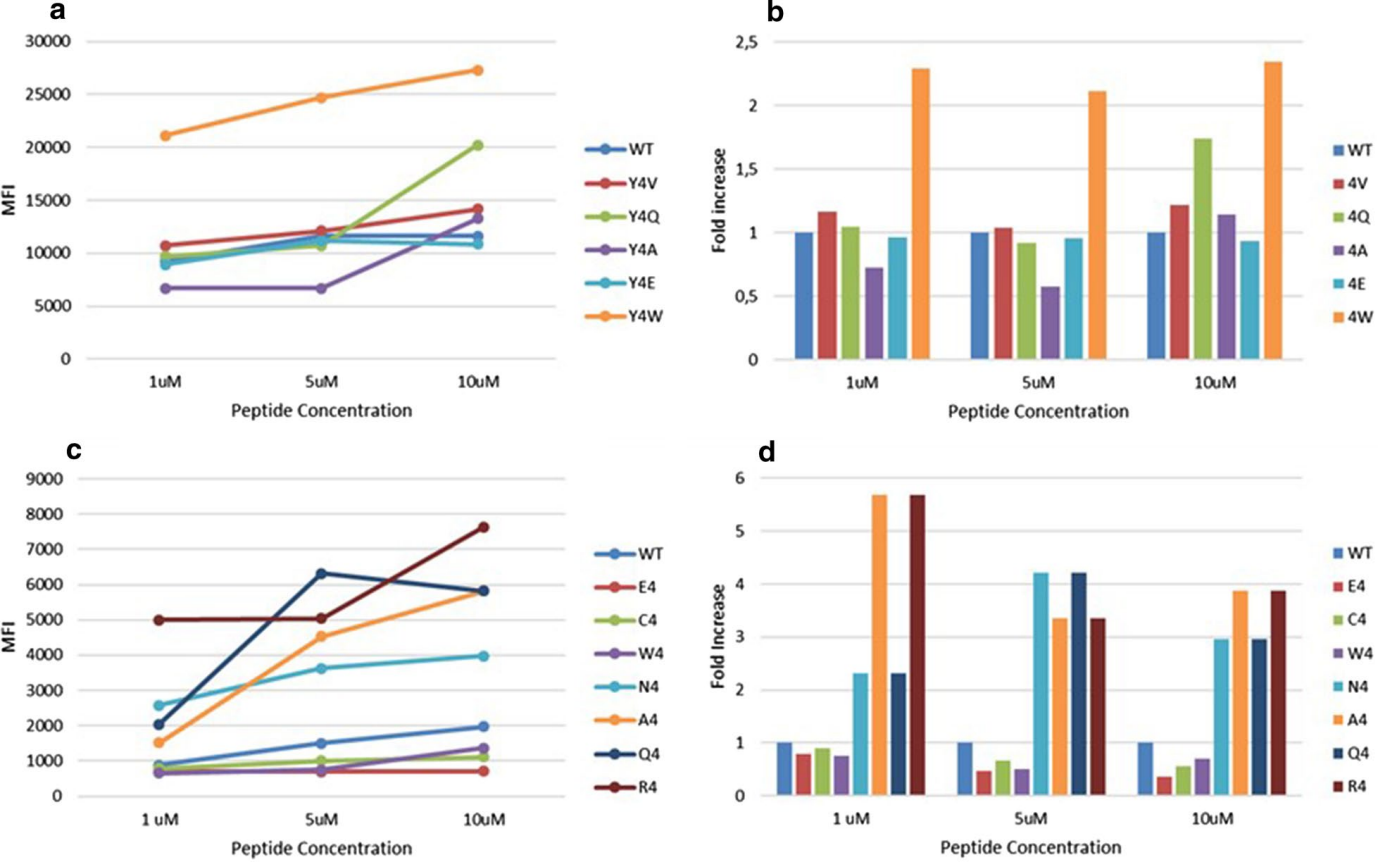
Binding affinity to the H2-Db molecule. Binding to H2-Db molecule was assessed in TAP-deficient RMA-S murine cells loaded with the indicated peptides. **a** Mean fluorescence intensity at flow cytometer indicates binding levels of HPV-E7 wt and heteroclitic peptides to the H2-Db molecule at the different concentrations. **b** Fold-increase (wt = 1) of the binding to the H2-Db molecule of each heteroclitic peptides at the different concentrations. **c** Mean fluorescence intensity at flow cytometer indicates binding levels of wt and heteroclitic Trp2 peptides to the H2-Db molecule at the different concentrations. **d** Fold-increase (wt = 1) of the binding to the H2-Db molecule of each heteroclitic peptides at the different concentrations

In order to experimentally confirm the binding of heteroclitic peptides to H-2 Db molecule, the H-2 Db positive RMA-S cell line was loaded with different concentrations (1 μM, 5 μM, 10 μM) of selected heteroclitic peptides modified in p4 with a predicted affinity higher than the wt peptide. The heteroclitic peptides of the E7 epitope, except for the Y4E substitution, showed a binding to the H-2Db stronger than the wt peptide, although the correlation with predicted binding was low. Indeed, the Y4W substitution, predicted to have a binding similar to the wt, showed the strongest binding in the assay (Fig. 7a, b). Similarly, most of the heteroclitic peptides of the Trp2 epitope showed a binding to the H-2Db stronger than the wt peptide, although the correlation with predicted binding was low. Also in this case, the substitutions D4W, D4C and D4E showed a binding lower than the wt although they were predicted to have a higher binding (Fig. 7c, d).

## Discussion

In the present study, we set up a simple strategy to design epitope variants with increased affinities for the H2-Db molecule. Heteroclitic peptides were designed with amino acid changes in one of the TCR-facing residues (p4) which does not have direct contacts with the H2-Db molecule. Such substitutions were predicted to impact on the peptide binding to the murine H2-Db molecule and, in particular, several of them induce an increased affinity. The two peptides used in the analysis, Trp2 and HPV-E7 peptides, are predicted to have dramatically different binding affinity to H2-Db molecule (6990 nM and 100 nM, respectively). Bioinformatics structural analyses confirm such a difference, showing that the structure of the residue in the anchor positions (p5) of the Trp2 peptide has a very poor compatibility with the H2-Db groove.

Molecular docking shows that, with the exception of the Y4Q, none of the other substitutions introduced in the p4 residue of the HPV-E7 peptide significantly changes the contact points of the E7 peptide with the H2-Db molecule. However, only the Y4V substitution, when docked into the H2-Db groove, results in a conformation of the TCR binding residues very similar to the wt peptide. Similarly, heteroclitic peptides of the Trp2 epitope show an identical pattern of contact points with the H2-Db of the wt peptide. Each heteroclitic peptide, when docked into the H2-Db groove, shows a substantial similarity compared to the wt, except for the D4R and the D4W substitutions.

The binding assay confirm that the heteroclitic peptides of the E7 epitope, except for the Y4E substitution, showed a binding to the H-2Db stronger than the wt peptide, although the correlation with predicted binding was rather low. Similarly, most of the heteroclitic peptides of the Trp2 epitope showed a binding to the H-2Db stronger than the wt peptide, although the correlation with predicted binding was low.

In conclusion, we demonstrated that modified heteroclitic TAAs can be improved by changing even one single side chain of the TCR-binding residues, achieving higher affinity to major histocompatibility class I (MHC-I) molecule. Therefore, such a strategy may be very effective for designing vaccine antigens with superior antigenic efficacy.

In this respect, a validation experiment is currently planned in an animal model, to confirm the in silico analysis described in the present study. This will establish the foundation for moving forward the concept into a human clinical trial.

## Data Availability

Data and material are available upon request.
